# Bis(2,3,5,6-tetra-2-pyridyl­pyrazine-κ^3^ *N* ^2^,*N* ^1^,*N* ^6^)nickel(II) dithio­cyanate dihydrate

**DOI:** 10.1107/S1600536810053419

**Published:** 2010-12-24

**Authors:** Noelia De la Pinta, M. Luz Fidalgo, José M. Ezpeleta, Roberto Cortés, Gotzon Madariaga

**Affiliations:** a Departamento de Química Inorgánica, Facultad de Ciencia y Tecnología, Universidad del País Vasco, Apdo. 644, E-48080 Bilbao, Spain; b Departamento de Química Inorgánica, Facultad de Farmacia, Universidad del País Vasco, Apdo. 450, E-01080 Vitoria, Spain; c Departamento de Física Aplicada II, Facultad de Farmacia, Universidad del País Vasco, Apdo. 450, E-01080 Vitoria, Spain; d Departamento de Física de la Materia Condensada, Facultad de Ciencia y Tecnología, Universidad del País Vasco, Apdo. 644, E-48080 Bilbao, Spain

## Abstract

In the title compound, [Ni(C_24_H_16_N_6_)_2_](NCS)_2_·2H_2_O, the central Ni^II^ ion is octahedrally coordinated by six N atoms of two tridentate 2,3,5,6-tetra-2-pyridyl­pyrazine ligands (tppz). Two thio­cyanate anions act as counter-ions and two water mol­ecules act as solvation agents. O—H⋯N hydrogen bonds are observed in the crystral structure.

## Related literature

For related structures including [*M*(II)(tppz)_2_]^2+^ cations, see: Ruminski & Kiplinger (1990 ▶); Arana *et al.* (1992 ▶); Lainé *et al.* (1995 ▶); Allis *et al.* (2004 ▶); Burkholder & Zubieta (2004 ▶); Haines *et al.* (2000 ▶). For the aplication of a [Co(II)(tppz)_2_]^2+^ complex as a homogeneous catalyst, see: Königstein & Bauer (1994 ▶, 1997 ▶).
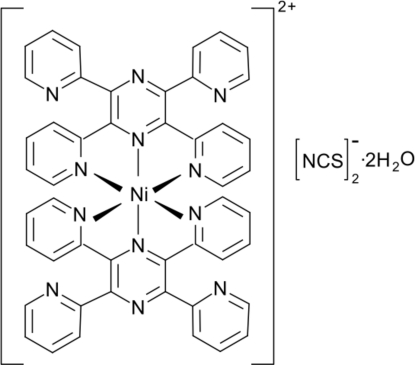


## Experimental

### 

#### Crystal data

[Ni(C_24_H_16_N_6_)_2_](NCS)_2_·2H_2_O
*M* *_r_* = 987.76
Monoclinic, 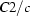

*a* = 17.9091 (4) Å
*b* = 13.6851 (2) Å
*c* = 19.4650 (4) Å
β = 106.161 (2)°
*V* = 4582.11 (15) Å^3^
*Z* = 4
Mo *K*α radiation
μ = 0.57 mm^−1^
*T* = 293 K
0.35 × 0.26 × 0.21 mm

#### Data collection

Oxford Diffraction Xcalibur Sapphire2 diffractometer
23806 measured reflections
7385 independent reflections
4946 reflections with *I* > 2σ(*I*)
*R*_int_ = 0.031

#### Refinement

*R*[*F* ^2^ > 2σ(*F* ^2^)] = 0.048
*wR*(*F* ^2^) = 0.123
*S* = 0.94
7385 reflections
320 parameters
4 restraints
H atoms treated by a mixture of independent and constrained refinement
Δρ_max_ = 0.56 e Å^−3^
Δρ_min_ = −0.43 e Å^−3^

### 

Data collection: *CrysAlis PRO* (Oxford Diffraction, 2007 ▶); cell refinement: *CrysAlis PRO*; data reduction: *CrysAlis PRO*; program(s) used to solve structure: *SIR97* (Altomare *et al.*, 1999 ▶); program(s) used to refine structure: *SHELXL97* (Sheldrick, 2008 ▶); molecular graphics: *Mercury* (Macrae *et al.*, 2006 ▶); software used to prepare material for publication: *WinGX* (Farrugia, 1999 ▶).

## Supplementary Material

Crystal structure: contains datablocks global, I. DOI: 10.1107/S1600536810053419/fj2379sup1.cif

Structure factors: contains datablocks I. DOI: 10.1107/S1600536810053419/fj2379Isup2.hkl

Additional supplementary materials:  crystallographic information; 3D view; checkCIF report

## Figures and Tables

**Table 1:** Hydrogen-bond geometry (Å, °)

*D*—H⋯*A*	*D*—H	H⋯*A*	*D*⋯*A*	*D*—H⋯*A*
O1*W*—H1*W*1⋯N9^i^	0.85 (2)	2.28 (3)	3.116 (4)	166 (3)
O1*W*—H1*W*2⋯N1^ii^	0.85 (4)	2.20 (3)	3.044 (4)	173 (4)

Symmetry codes: (i) 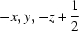; (ii) 
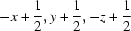
.

**Fig. 1.**
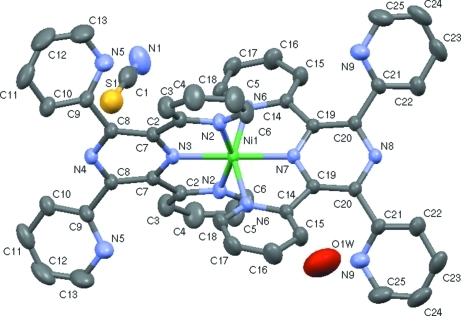
Asymmetric unit of [Ni(C24H16N6)2](NCS)2.2H2O showing the most relevant labels. H atoms excluded for clarity. Ellipsoids drawn at 50% probability level.

**Fig. 2.**
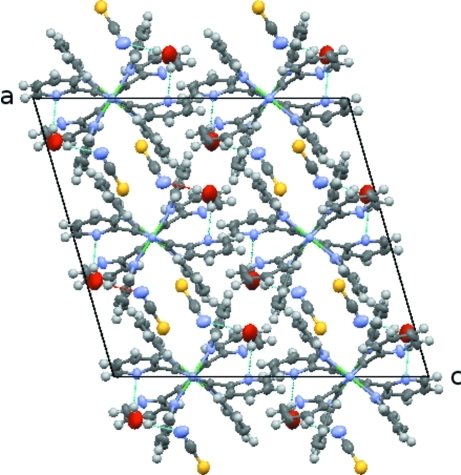
Projection of [Ni(C24H16N6)2](NCS)2.2H2O packing along the b axis showing the link between pyridyl and NCS groups through the N1—O1W—N9 H-bonds.

## supplementary crystallographic information

### Comment

The combination of divalent cations of the second half of the transition series with the ligand tppz, gives coordination cations of the type [*M*^II^(tppz)_2_]^2+^, where the terminal nitrogen atoms of one extreme of the tppz ligand are directed towards the metallic atoms and the corresponding N atoms of the other extreme remain uncoordinated (Lainé *et al.*, 1995). These tppz cations are part of diferent coordination compounds (Allis *et al.*, 2004, Burkholder & Zubieta, 2004, Ruminski *et al.*, 1990, Haines *et al.*, 2000, Arana *et al.*, 1992, Köningstein *et al.*, 1997,, 1994). We here report the crystal structure of a new compound, [Ni(C_24_H_16_N_6_)_2_](NCS)_2_.2H_2_O, which is made up of Ni^II^ cations coordinated to six nitrogen atoms of two tridentate tppz ligands. These monomeric entities reach the neutrality with two thiocianate anions and two molecules of water act as solvent agents. The nitrogen atoms of each tppz, coordinated to the cation, are in the same plane. The Ni^II^ cation has a distorted octahedral environment, in which the bonds to the two pyrazine nitrogen atoms (Ni1—N3 and Ni1—N7) are significantly shorter than the bonds to the pyridyl nitrogen atoms (Ni1—N2 and Ni1—N6). The N—Ni1—N angles involving the atoms of the equatorial plane with respect to the short axis differ remarkably from the ideal octahedral values, the Ni1 atom not deviating significantly [0.0006 (2) Å] from the average plane.

The individual pyridyl rings are planar (maximum average displacement with respect to the plane of the ring, 0.011 Å), while the two pyrazine rings are significantly puckered. Nitrogen atoms of the non-coordinated pyridyl rings of the two tppz ligands point to the ligand metalated side instead of the free nitrogen atom of the pyrazine ring. These structural features of the coordinated tppz ligand would account for its tendency to adopt bis-chelation in this type of complexes. Weak π-π interactions appear to occur due to overlap between the pyridyl rings labelled by N5 and N9^iii^ (iii= -*x*,-1 + *y*,1/2 - *z*) with a distance between the ring centroids of 3.7713 (12) Å.

The crystal packing of the bulky building block units leaves cavities of 116 Å^3^ where water molecules and NCS anions are located (Fig. 2). The low density of the material reflects this open structure. Most relevant H bonds are listed in Table 1.

### Experimental

Crystals of [Ni(C_24_H_16_N_6_)_2_](NCS)_2_.2H_2_O were prepared by mixing an acetonitrile solution (10 ml) of NiCl_2_.6H_2_O (112 mg, 0.50 mmol) and another acetonitrile solution (10 ml) of 2,3,5,6-tetra-2-pyridylpirazine (97.1 mg, 0.25 mmol). After a vigorous stirring of about 30 minutes at a temperature of 303 K, an aqua/acetonitrile (50%) solution (10 ml) of sodium thiocyanate (101.3 mg, 1.25 mmol) was added. The resultant solution was stirred at 313 K for 25 minutes and at room temperature for the following two days. Then the precipitate that did form was filtered off. Finally, the resulting solution was maintained at room temperature until prismatic green crystals were formed by slow evaporation. These crystals were found to be stable to X-ray exposure.

### Refinement

Structure solution by direct methods in the space group *C*2/*c*, followed by refinement, based on F^2^, of atomic coordinates and anisotropic displacement parameters, was performed using the programs *SIR97* (Altomare *et al.*, 1999) and *SHELXL97* (Sheldrick, 2008) successively. H atoms bonded to C atoms were found in successive difference Fourier maps and refined using a riding model, with C—H = 0.93 Å and with *U*_iso_(H) = 1.5*U*_eq_(C). H atoms of O1W (see Fig. 1 for labelling) were approximately located in a difference Fourier map. During the refinement their positions were tightly restrained to the ideal geometry [O—H=0.85 (1) Å, H—O—H=107 (3)°] with *U*_iso_(H) = 1.5*U*_eq_(O). Also the distances H1W1—N9^i^ (i= -*x*, *y*, -*z* + 1/2 and H1W2—N1^ii^ (ii= -*x* + 1/2, *y* + 1/2,-*z* + 1/2 were forced to be equal (within 0.02 Å). The highest residual electron density is 0.02 Å from atom Ni1 and the deepest hole is 0.65 Å from atom S1.

### Crystal data

**Table:**

[Ni(C_24_H_16_N_6_)_2_](NCS)_2_·2H_2_O	*F*(000) = 2040
*M**_r_* = 987.76	*D*_x_ = 1.432 Mg m^−^^3^
Monoclinic, *C*2/*c*	Mo *K*α radiation, λ = 0.71073 Å
Hall symbol: -C 2yc	Cell parameters from 9376 reflections
*a* = 17.9091 (4) Å	θ = 3.0–32.2°
*b* = 13.6851 (2) Å	µ = 0.57 mm^−^^1^
*c* = 19.4650 (4) Å	*T* = 293 K
β = 106.161 (2)°	Prism, green
*V* = 4582.11 (15) Å^3^	0.35 × 0.26 × 0.21 mm
*Z* = 4	

### Data collection

**Table:**

Oxford Diffraction Xcalibur Sapphire2 window diffractometer	4946 reflections with *I* > 2σ(*I*)
Radiation source: sealed X-ray tube	*R*_int_ = 0.031
graphite	θ_max_ = 32.2°, θ_min_ = 3.0°
Detector resolution: 8.3504 pixels mm^-1^	*h* = −25→21
ω scans	*k* = −18→20
23806 measured reflections	*l* = −28→29
7385 independent reflections	

### Refinement

**Table:**

Refinement on *F*^2^	Primary atom site location: structure-invariant direct methods
Least-squares matrix: full	Secondary atom site location: difference Fourier map
*R*[*F*^2^ > 2σ(*F*^2^)] = 0.048	Hydrogen site location: inferred from neighbouring sites
*wR*(*F*^2^) = 0.123	H atoms treated by a mixture of independent and constrained refinement
*S* = 0.94	*w* = 1/[σ^2^(*F*_o_^2^) + (0.0771*P*)^2^] where *P* = (*F*_o_^2^ + 2*F*_c_^2^)/3
7385 reflections	(Δ/σ)_max_ < 0.001
320 parameters	Δρ_max_ = 0.56 e Å^−^^3^
4 restraints	Δρ_min_ = −0.43 e Å^−^^3^

### Special details

**Table:**

Geometry. All s.u.'s (except the s.u. in the dihedral angle between two l.s. planes) are estimated using the full covariance matrix. The cell s.u.'s are taken into account individually in the estimation of s.u.'s in distances, angles and torsion angles; correlations between s.u.'s in cell parameters are only used when they are defined by crystal symmetry. An approximate (isotropic) treatment of cell s.u.'s is used for estimating s.u.'s involving l.s. planes.
Refinement. Refinement of *F*^2^ against ALL reflections. The weighted *R*-factor *wR* and goodness of fit *S* are based on *F*^2^, conventional *R*-factors *R* are based on *F*, with *F* set to zero for negative *F*^2^. The threshold expression of *F*^2^ > 2σ(*F*^2^) is used only for calculating *R*-factors(gt) *etc*. and is not relevant to the choice of reflections for refinement. *R*-factors based on *F*^2^ are statistically about twice as large as those based on *F*, and *R*- factors based on ALL data will be even larger.

### Fractional atomic coordinates and isotropic or equivalent isotropic displacement parameters (Å^2^)

**Table:**

	*x*	*y*	*z*	*U*_iso_*/*U*_eq_	
Ni1	0	0.310758 (18)	0.25	0.03035 (9)	
N1	0.30210 (13)	0.08495 (16)	0.16393 (13)	0.0748 (6)	
C1	0.24787 (15)	0.05532 (16)	0.17858 (12)	0.0613 (6)	
S1	0.17309 (4)	0.01597 (5)	0.20104 (4)	0.07129 (18)	
N2	0.09989 (8)	0.27794 (9)	0.33345 (7)	0.0357 (3)	
N3	0	0.16447 (12)	0.25	0.0275 (4)	
N4	0	−0.03295 (12)	0.25	0.0297 (4)	
N5	0.09070 (11)	−0.00169 (11)	0.43264 (8)	0.0519 (4)	
N6	0.07402 (8)	0.34349 (10)	0.18698 (7)	0.0346 (3)	
N7	0	0.45723 (12)	0.25	0.0282 (4)	
N8	0	0.65503 (12)	0.25	0.0287 (4)	
N9	0.01006 (10)	0.61824 (11)	0.07513 (8)	0.0450 (4)	
C2	0.11628 (9)	0.18165 (10)	0.34250 (8)	0.0318 (3)	
C3	0.18842 (10)	0.14902 (13)	0.38158 (11)	0.0451 (4)	
H3	0.1988	0.0824	0.3867	0.068*	
C4	0.24504 (12)	0.21619 (16)	0.41296 (13)	0.0603 (6)	
H4	0.2941	0.1955	0.4393	0.09*	
C5	0.22787 (13)	0.31443 (15)	0.40478 (13)	0.0631 (6)	
H5	0.2649	0.3611	0.426	0.095*	
C6	0.15514 (12)	0.34205 (14)	0.36470 (11)	0.0500 (5)	
H6	0.1438	0.4084	0.359	0.075*	
C7	0.05337 (9)	0.11697 (10)	0.30076 (8)	0.0279 (3)	
C8	0.04567 (8)	0.01515 (10)	0.30544 (8)	0.0284 (3)	
C9	0.08377 (9)	−0.04437 (11)	0.36910 (8)	0.0327 (3)	
C10	0.10704 (10)	−0.13895 (12)	0.36186 (10)	0.0399 (4)	
H10	0.0997	−0.1664	0.3168	0.06*	
C11	0.14136 (11)	−0.19211 (14)	0.42277 (12)	0.0538 (5)	
H11	0.1583	−0.2557	0.4197	0.081*	
C12	0.14968 (14)	−0.14892 (19)	0.48751 (13)	0.0686 (7)	
H12	0.1729	−0.1827	0.5295	0.103*	
C13	0.12351 (16)	−0.05524 (19)	0.49030 (11)	0.0702 (7)	
H13	0.129	−0.0275	0.535	0.105*	
C14	0.08424 (9)	0.43995 (10)	0.17709 (8)	0.0299 (3)	
C15	0.14292 (10)	0.47369 (12)	0.15021 (10)	0.0405 (4)	
H15	0.1486	0.5402	0.1433	0.061*	
C16	0.19345 (11)	0.40681 (14)	0.13364 (11)	0.0494 (5)	
H16	0.2333	0.4279	0.1151	0.074*	
C17	0.18406 (11)	0.30917 (13)	0.14488 (10)	0.0452 (4)	
H17	0.2184	0.2634	0.1355	0.068*	
C18	0.12336 (11)	0.28013 (12)	0.17007 (9)	0.0413 (4)	
H18	0.116	0.2137	0.1757	0.062*	
C19	0.03442 (9)	0.50448 (10)	0.20708 (8)	0.0282 (3)	
C20	0.02362 (9)	0.60616 (10)	0.20014 (8)	0.0284 (3)	
C21	0.03384 (9)	0.66321 (11)	0.13860 (8)	0.0314 (3)	
C22	0.06215 (11)	0.75722 (12)	0.14626 (10)	0.0443 (4)	
H22	0.078	0.7865	0.1911	0.066*	
C23	0.06656 (12)	0.80718 (14)	0.08575 (12)	0.0551 (5)	
H23	0.0854	0.8708	0.0894	0.083*	
C24	0.04322 (13)	0.76275 (17)	0.02107 (13)	0.0601 (6)	
H24	0.0457	0.7952	−0.0202	0.09*	
C25	0.01589 (13)	0.66902 (18)	0.01790 (11)	0.0581 (5)	
H25	0.0004	0.6387	−0.0266	0.087*	
O1W	0.15572 (18)	0.5266 (3)	0.47152 (14)	0.1363 (10)	
H1W1	0.1090 (10)	0.546 (3)	0.465 (2)	0.204*	
H1W2	0.171 (2)	0.545 (4)	0.4359 (16)	0.204*	

### Atomic displacement parameters (Å^2^)

**Table:**

	*U*^11^	*U*^22^	*U*^33^	*U*^12^	*U*^13^	*U*^23^
Ni1	0.04230 (17)	0.01832 (13)	0.03170 (15)	0	0.01241 (12)	0
N1	0.0666 (13)	0.0573 (12)	0.1034 (17)	0.0104 (10)	0.0287 (12)	0.0182 (11)
C1	0.0809 (16)	0.0431 (11)	0.0536 (12)	0.0199 (11)	0.0083 (11)	0.0009 (9)
S1	0.0804 (4)	0.0647 (4)	0.0714 (4)	0.0071 (3)	0.0255 (3)	0.0001 (3)
N2	0.0454 (8)	0.0242 (6)	0.0360 (7)	−0.0069 (5)	0.0086 (6)	−0.0026 (5)
N3	0.0302 (9)	0.0223 (8)	0.0298 (9)	0	0.0081 (7)	0
N4	0.0345 (9)	0.0217 (8)	0.0317 (9)	0	0.0073 (7)	0
N5	0.0743 (11)	0.0435 (9)	0.0326 (8)	−0.0023 (8)	0.0063 (8)	0.0012 (6)
N6	0.0471 (8)	0.0250 (6)	0.0346 (7)	0.0031 (5)	0.0160 (6)	−0.0022 (5)
N7	0.0346 (9)	0.0222 (8)	0.0300 (9)	0	0.0127 (7)	0
N8	0.0363 (9)	0.0219 (8)	0.0295 (9)	0	0.0117 (7)	0
N9	0.0563 (9)	0.0464 (9)	0.0335 (7)	−0.0011 (7)	0.0146 (7)	0.0023 (6)
C2	0.0375 (8)	0.0254 (7)	0.0310 (7)	−0.0036 (6)	0.0072 (6)	−0.0017 (6)
C3	0.0373 (9)	0.0359 (9)	0.0567 (11)	−0.0003 (7)	0.0039 (8)	−0.0016 (8)
C4	0.0395 (10)	0.0553 (12)	0.0736 (15)	−0.0079 (9)	−0.0048 (10)	−0.0044 (11)
C5	0.0536 (12)	0.0478 (12)	0.0753 (15)	−0.0209 (9)	−0.0029 (11)	−0.0068 (10)
C6	0.0578 (12)	0.0305 (8)	0.0548 (11)	−0.0128 (8)	0.0042 (9)	−0.0058 (8)
C7	0.0323 (7)	0.0235 (7)	0.0276 (7)	−0.0003 (5)	0.0078 (6)	−0.0007 (5)
C8	0.0315 (7)	0.0231 (7)	0.0296 (7)	0.0012 (5)	0.0070 (6)	0.0010 (5)
C9	0.0342 (8)	0.0276 (7)	0.0329 (8)	−0.0031 (6)	0.0038 (6)	0.0044 (6)
C10	0.0397 (9)	0.0313 (8)	0.0464 (10)	0.0011 (7)	0.0081 (7)	0.0075 (7)
C11	0.0410 (10)	0.0405 (10)	0.0753 (14)	0.0034 (8)	0.0087 (10)	0.0251 (10)
C12	0.0684 (14)	0.0713 (15)	0.0527 (13)	−0.0042 (12)	−0.0056 (11)	0.0324 (12)
C13	0.0956 (18)	0.0725 (16)	0.0327 (10)	−0.0073 (13)	0.0019 (11)	0.0079 (10)
C14	0.0380 (8)	0.0244 (7)	0.0287 (7)	0.0027 (6)	0.0117 (6)	−0.0017 (5)
C15	0.0464 (10)	0.0293 (8)	0.0521 (10)	−0.0007 (7)	0.0242 (8)	−0.0010 (7)
C16	0.0466 (10)	0.0456 (10)	0.0650 (12)	−0.0007 (8)	0.0306 (9)	−0.0082 (9)
C17	0.0485 (10)	0.0391 (9)	0.0523 (10)	0.0080 (7)	0.0208 (9)	−0.0096 (8)
C18	0.0550 (10)	0.0281 (8)	0.0444 (9)	0.0078 (7)	0.0197 (8)	−0.0051 (7)
C19	0.0347 (7)	0.0238 (7)	0.0281 (7)	0.0002 (5)	0.0121 (6)	0.0002 (5)
C20	0.0346 (7)	0.0222 (6)	0.0300 (7)	0.0002 (5)	0.0119 (6)	0.0021 (5)
C21	0.0361 (8)	0.0274 (7)	0.0344 (8)	0.0043 (6)	0.0158 (6)	0.0061 (6)
C22	0.0557 (11)	0.0321 (8)	0.0523 (11)	−0.0023 (7)	0.0267 (9)	0.0037 (7)
C23	0.0629 (13)	0.0405 (10)	0.0730 (14)	0.0026 (9)	0.0372 (11)	0.0207 (10)
C24	0.0595 (13)	0.0712 (14)	0.0579 (13)	0.0129 (10)	0.0300 (10)	0.0339 (11)
C25	0.0623 (13)	0.0775 (15)	0.0352 (10)	0.0049 (11)	0.0147 (9)	0.0107 (9)
O1W	0.144 (2)	0.167 (3)	0.108 (2)	0.035 (2)	0.0506 (18)	−0.0268 (18)

### Geometric parameters (Å, °)

**Table:**

Ni1—N3	2.0019 (17)	C6—H6	0.93
Ni1—N7	2.0045 (17)	C7—C8	1.4057 (19)
Ni1—N6^i^	2.0899 (14)	C8—C9	1.481 (2)
Ni1—N6	2.0899 (14)	C9—C10	1.379 (2)
Ni1—N2^i^	2.1030 (14)	C10—C11	1.381 (2)
Ni1—N2	2.1030 (14)	C10—H10	0.93
N1—C1	1.159 (3)	C11—C12	1.362 (3)
C1—S1	1.613 (3)	C11—H11	0.93
N2—C6	1.336 (2)	C12—C13	1.371 (4)
N2—C2	1.3509 (19)	C12—H12	0.93
N3—C7^i^	1.3366 (16)	C13—H13	0.93
N3—C7	1.3366 (16)	C14—C15	1.378 (2)
N4—C8	1.3325 (16)	C14—C19	1.485 (2)
N4—C8^i^	1.3325 (16)	C15—C16	1.387 (2)
N5—C13	1.332 (3)	C15—H15	0.93
N5—C9	1.342 (2)	C16—C17	1.372 (3)
N6—C18	1.343 (2)	C16—H16	0.93
N6—C14	1.3538 (19)	C17—C18	1.370 (3)
N7—C19	1.3353 (16)	C17—H17	0.93
N7—C19^i^	1.3353 (16)	C18—H18	0.93
N8—C20	1.3404 (17)	C19—C20	1.4062 (19)
N8—C20^i^	1.3404 (17)	C20—C21	1.483 (2)
N9—C21	1.339 (2)	C21—C22	1.376 (2)
N9—C25	1.342 (2)	C22—C23	1.383 (3)
C2—C3	1.378 (2)	C22—H22	0.93
C2—C7	1.484 (2)	C23—C24	1.355 (3)
C3—C4	1.379 (3)	C23—H23	0.93
C3—H3	0.93	C24—C25	1.368 (3)
C4—C5	1.378 (3)	C24—H24	0.93
C4—H4	0.93	C25—H25	0.93
C5—C6	1.372 (3)	O1W—H1W1	0.85 (2)
C5—H5	0.93	O1W—H1W2	0.85 (4)
			
N3—Ni1—N7	180	N5—C9—C10	123.34 (15)
N3—Ni1—N6^i^	102.38 (4)	N5—C9—C8	115.76 (14)
N7—Ni1—N6^i^	77.62 (4)	C10—C9—C8	120.84 (14)
N3—Ni1—N6	102.38 (4)	C9—C10—C11	118.80 (18)
N7—Ni1—N6	77.62 (4)	C9—C10—H10	120.6
N6^i^—Ni1—N6	155.25 (7)	C11—C10—H10	120.6
N3—Ni1—N2^i^	77.67 (4)	C12—C11—C10	118.28 (19)
N7—Ni1—N2^i^	102.33 (4)	C12—C11—H11	120.9
N6^i^—Ni1—N2^i^	87.53 (5)	C10—C11—H11	120.9
N6—Ni1—N2^i^	97.74 (5)	C11—C12—C13	119.46 (18)
N3—Ni1—N2	77.67 (4)	C11—C12—H12	120.3
N7—Ni1—N2	102.33 (4)	C13—C12—H12	120.3
N6^i^—Ni1—N2	97.74 (5)	N5—C13—C12	123.8 (2)
N6—Ni1—N2	87.53 (5)	N5—C13—H13	118.1
N2^i^—Ni1—N2	155.34 (7)	C12—C13—H13	118.1
N1—C1—S1	178.4 (2)	N6—C14—C15	121.91 (14)
C6—N2—C2	118.34 (15)	N6—C14—C19	113.83 (13)
C6—N2—Ni1	125.08 (12)	C15—C14—C19	123.72 (14)
C2—N2—Ni1	114.60 (10)	C14—C15—C16	118.90 (16)
C7^i^—N3—C7	121.80 (17)	C14—C15—H15	120.5
C7^i^—N3—Ni1	119.10 (9)	C16—C15—H15	120.5
C7—N3—Ni1	119.10 (9)	C17—C16—C15	119.27 (17)
C8—N4—C8^i^	120.79 (17)	C17—C16—H16	120.4
C13—N5—C9	116.30 (17)	C15—C16—H16	120.4
C18—N6—C14	118.07 (14)	C18—C17—C16	118.97 (16)
C18—N6—Ni1	125.01 (12)	C18—C17—H17	120.5
C14—N6—Ni1	115.18 (10)	C16—C17—H17	120.5
C19—N7—C19^i^	122.07 (18)	N6—C18—C17	122.84 (16)
C19—N7—Ni1	118.97 (9)	N6—C18—H18	118.6
C19^i^—N7—Ni1	118.97 (9)	C17—C18—H18	118.6
C20—N8—C20^i^	120.13 (17)	N7—C19—C20	117.66 (13)
C21—N9—C25	116.73 (17)	N7—C19—C14	112.97 (13)
N2—C2—C3	121.62 (14)	C20—C19—C14	129.22 (13)
N2—C2—C7	113.95 (13)	N8—C20—C19	119.14 (13)
C3—C2—C7	124.12 (14)	N8—C20—C21	117.19 (13)
C2—C3—C4	119.29 (17)	C19—C20—C21	123.62 (13)
C2—C3—H3	120.4	N9—C21—C22	122.87 (15)
C4—C3—H3	120.4	N9—C21—C20	115.10 (14)
C5—C4—C3	119.08 (19)	C22—C21—C20	121.96 (15)
C5—C4—H4	120.5	C21—C22—C23	118.46 (18)
C3—C4—H4	120.5	C21—C22—H22	120.8
C6—C5—C4	118.71 (17)	C23—C22—H22	120.8
C6—C5—H5	120.6	C24—C23—C22	119.57 (19)
C4—C5—H5	120.6	C24—C23—H23	120.2
N2—C6—C5	122.94 (18)	C22—C23—H23	120.2
N2—C6—H6	118.5	C23—C24—C25	118.46 (18)
C5—C6—H6	118.5	C23—C24—H24	120.8
N3—C7—C8	117.85 (13)	C25—C24—H24	120.8
N3—C7—C2	113.09 (13)	N9—C25—C24	123.9 (2)
C8—C7—C2	128.96 (13)	N9—C25—H25	118
N4—C8—C7	119.10 (13)	C24—C25—H25	118
N4—C8—C9	116.22 (13)	H1W1—O1W—H1W2	108 (4)
C7—C8—C9	124.66 (13)		
			
N3—Ni1—N2—C6	167.18 (16)	C8^i^—N4—C8—C7	−8.67 (10)
N7—Ni1—N2—C6	−12.82 (16)	C8^i^—N4—C8—C9	170.04 (15)
N6^i^—Ni1—N2—C6	−91.77 (16)	N3—C7—C8—N4	17.43 (19)
N6—Ni1—N2—C6	63.94 (16)	C2—C7—C8—N4	−158.78 (13)
N2^i^—Ni1—N2—C6	167.18 (16)	N3—C7—C8—C9	−161.16 (13)
N3—Ni1—N2—C2	3.52 (10)	C2—C7—C8—C9	22.6 (2)
N7—Ni1—N2—C2	−176.48 (10)	C13—N5—C9—C10	1.5 (3)
N6^i^—Ni1—N2—C2	104.58 (11)	C13—N5—C9—C8	178.73 (18)
N6—Ni1—N2—C2	−99.71 (11)	N4—C8—C9—N5	−143.30 (14)
N2^i^—Ni1—N2—C2	3.52 (10)	C7—C8—C9—N5	35.3 (2)
N6^i^—Ni1—N3—C7^i^	89.60 (8)	N4—C8—C9—C10	34.0 (2)
N6—Ni1—N3—C7^i^	−90.40 (8)	C7—C8—C9—C10	−147.32 (16)
N2^i^—Ni1—N3—C7^i^	4.94 (8)	N5—C9—C10—C11	−2.0 (3)
N2—Ni1—N3—C7^i^	−175.06 (8)	C8—C9—C10—C11	−179.12 (16)
N6^i^—Ni1—N3—C7	−90.40 (8)	C9—C10—C11—C12	0.9 (3)
N6—Ni1—N3—C7	89.60 (8)	C10—C11—C12—C13	0.5 (3)
N2^i^—Ni1—N3—C7	−175.06 (8)	C9—N5—C13—C12	0.1 (4)
N2—Ni1—N3—C7	4.94 (8)	C11—C12—C13—N5	−1.0 (4)
N3—Ni1—N6—C18	−16.09 (14)	C18—N6—C14—C15	0.3 (2)
N7—Ni1—N6—C18	163.91 (14)	Ni1—N6—C14—C15	166.03 (13)
N6^i^—Ni1—N6—C18	163.91 (14)	C18—N6—C14—C19	−171.54 (14)
N2^i^—Ni1—N6—C18	−95.09 (14)	Ni1—N6—C14—C19	−5.83 (16)
N2—Ni1—N6—C18	60.72 (14)	N6—C14—C15—C16	−0.8 (3)
N3—Ni1—N6—C14	179.33 (10)	C19—C14—C15—C16	170.27 (16)
N7—Ni1—N6—C14	−0.67 (10)	C14—C15—C16—C17	−0.5 (3)
N6^i^—Ni1—N6—C14	−0.67 (10)	C15—C16—C17—C18	2.1 (3)
N2^i^—Ni1—N6—C14	100.33 (11)	C14—N6—C18—C17	1.4 (3)
N2—Ni1—N6—C14	−103.87 (11)	Ni1—N6—C18—C17	−162.77 (14)
N6^i^—Ni1—N7—C19	−171.86 (8)	C16—C17—C18—N6	−2.6 (3)
N6—Ni1—N7—C19	8.14 (8)	C19^i^—N7—C19—C20	−9.30 (10)
N2^i^—Ni1—N7—C19	−87.20 (8)	Ni1—N7—C19—C20	170.70 (10)
N2—Ni1—N7—C19	92.80 (8)	C19^i^—N7—C19—C14	166.59 (14)
N6^i^—Ni1—N7—C19^i^	8.14 (8)	Ni1—N7—C19—C14	−13.41 (14)
N6—Ni1—N7—C19^i^	−171.86 (8)	N6—C14—C19—N7	12.20 (18)
N2^i^—Ni1—N7—C19^i^	92.80 (8)	C15—C14—C19—N7	−159.49 (14)
N2—Ni1—N7—C19^i^	−87.20 (8)	N6—C14—C19—C20	−172.49 (15)
C6—N2—C2—C3	−1.4 (2)	C15—C14—C19—C20	15.8 (3)
Ni1—N2—C2—C3	163.47 (14)	C20^i^—N8—C20—C19	−9.49 (10)
C6—N2—C2—C7	−175.20 (15)	C20^i^—N8—C20—C21	168.10 (15)
Ni1—N2—C2—C7	−10.38 (17)	N7—C19—C20—N8	19.1 (2)
N2—C2—C3—C4	0.8 (3)	C14—C19—C20—N8	−156.03 (14)
C7—C2—C3—C4	174.00 (18)	N7—C19—C20—C21	−158.34 (13)
C2—C3—C4—C5	0.4 (3)	C14—C19—C20—C21	26.5 (2)
C3—C4—C5—C6	−0.9 (4)	C25—N9—C21—C22	0.8 (3)
C2—N2—C6—C5	0.8 (3)	C25—N9—C21—C20	177.73 (16)
Ni1—N2—C6—C5	−162.32 (18)	N8—C20—C21—N9	−140.33 (14)
C4—C5—C6—N2	0.4 (4)	C19—C20—C21—N9	37.1 (2)
C7^i^—N3—C7—C8	−8.50 (10)	N8—C20—C21—C22	36.6 (2)
Ni1—N3—C7—C8	171.50 (10)	C19—C20—C21—C22	−145.93 (17)
C7^i^—N3—C7—C2	168.30 (14)	N9—C21—C22—C23	−0.3 (3)
Ni1—N3—C7—C2	−11.70 (14)	C20—C21—C22—C23	−177.02 (16)
N2—C2—C7—N3	14.24 (18)	C21—C22—C23—C24	−0.1 (3)
C3—C2—C7—N3	−159.43 (15)	C22—C23—C24—C25	0.0 (3)
N2—C2—C7—C8	−169.40 (15)	C21—N9—C25—C24	−1.0 (3)
C3—C2—C7—C8	16.9 (3)	C23—C24—C25—N9	0.6 (3)

Symmetry codes: (i) −*x*, *y*, −*z*+1/2.

### Hydrogen-bond geometry (Å, °)

**Table:**

*D*—H···*A*	*D*—H	H···*A*	*D*···*A*	*D*—H···*A*
O1W—H1W1···N9^ii^	0.85 (2)	2.28 (3)	3.116 (4)	166 (3)
O1W—H1W2···N1^iii^	0.85 (4)	2.20 (3)	3.044 (4)	173 (4)
C3—H3···N5	0.93	2.61	3.045 (3)	109

Symmetry codes: (ii) −*x*, *y*, −*z*+1/2; (iii) −*x*+1/2, *y*+1/2, −*z*+1/2.

## References

[bb1] Allis, D. G., Burkholder, E. & Zubieta, J. (2004). *Polyhedron*, **23**, 1145–1152.

[bb2] Altomare, A., Burla, M. C., Camalli, M., Cascarano, G. L., Giacovazzo, C., Guagliardi, A., Moliterni, A. G. G., Polidori, G. & Spagna, R. (1999). *J. Appl. Cryst.* **32**, 115–119.

[bb3] Arana, C., Yan, S., Keshavarz, K. M., Potes, K. T. & Abruña, H. D. (1992). *Inorg. Chem.* **31**, 3680–3682.

[bb4] Burkholder, E. & Zubieta, J. (2004). *Inorg. Chim. Acta*, **357**, 1229–1235.

[bb5] Farrugia, L. J. (1999). *J. Appl. Cryst.* **32**, 837–838.

[bb6] Haines, R. I., Hutchings, D. R. & Strickland, D. W. (2000). *Inorganic Reaction Mechanisms (Amsterdam)*, **2**, 223–233.

[bb7] Königstein, C. & Bauer, R. (1994). *Hydrogen Energy Prog. X, Proc. World Hydrogen Energy Conf.* 10th, **2**, 717–725.

[bb8] Königstein, C. & Bauer, R. (1997). *Int. Journal Hydrogen Energy*, **22**, 471–474.

[bb9] Lainé, P., Gourdon, A. & Launay, J. P. (1995). *Inorg. Chem.* **34**, 5156–5165.

[bb10] Macrae, C. F., Edgington, P. R., McCabe, P., Pidcock, E., Shields, G. P., Taylor, R., Towler, M. & van de Streek, J. (2006). *J. Appl. Cryst.* **39**, 453–457.

[bb11] Oxford Diffraction (2007). *CrysAlis PRO* Oxford Diffraction Ltd, Abingdon, England.

[bb12] Ruminski, R. R. & Kiplinger, J. L. (1990). *Inorg. Chem.* **29**, 4581–4584.

[bb13] Sheldrick, G. M. (2008). *Acta Cryst.* A**64**, 112–122. 10.1107/S0108767307043930 18156677

